# The Dual Role of Macrophages in MIRI and MI by Immunity and Inflammation: Damage, Repair, Crosstalk, and Therapy

**DOI:** 10.1155/mi/9912957

**Published:** 2025-10-06

**Authors:** Zhilin Miao, Yamki Leung, Yuqing Fu, Li Luo, Quan Liu, Jianbo Liao, Jiatang Xu, Qingyang Song, Suiqing Huang, Zhongkai Wu

**Affiliations:** ^1^Department of Cardiac Surgery, The First Affiliated Hospital of Sun Yat-sen University, Guangzhou 510080, Guangdong, China; ^2^Organ Transplant Center, The First Affiliated Hospital of Sun Yat-sen University, Guangzhou 510080, Guangdong, China; ^3^Department of Cardiology, The Eighth Affiliated Hospital of Sun Yat-sen University, Shenzhen, Guangdong, China

**Keywords:** heart, immune, inflammation, ischemia reperfusion injury, macrophage, myocardial infarction

## Abstract

The global prevalence of coronary artery disease (CAD) continues to escalate globally. A substantial proportion of CAD patients develop myocardial ischemic injury or myocardial infarction (MI), while reperfusion therapy paradoxically induces myocardial ischemia/reperfusion injury (MIRI). Tissue-resident and recruited macrophages critically orchestrate cardiac inflammation resolution and repair-remodeling processes. Pathogenically, MIRI features early explosive inflammation with secondary reperfusion injury, whereas MI progresses from acute inflammation to reparative fibrosis. We highlight two pivotal macrophage subsets—C─C chemokine receptor type 2 (CCR2)^high^ macrophages dominating early MI phases and triggering receptor expressed on myeloid cells 2 (TREM2)^high^ macrophages prevailing in late stages—exploring their distinct roles in both MI and MIRI. This includes examining macrophage crosstalk with neutrophils and other immune-inflammatory cells. Finally, we discuss macrophage-targeted therapies encompassing anti-inflammatory modulation, exosome-mediated delivery, and stem cell interventions to mitigate cardiac injury progression.

## 1. Myocardial Ischemia/Reperfusion Injury (MIRI) and Myocardial Infarction (MI)

Globally, coronary artery disease (CAD) has the highest mortality rate [1], with over three million annual cases of acute ST-segment elevation MI where timely reperfusion therapy is the most effective treatment [2]. While restoring blood supply, reperfusion triggers pathological reactions—including peroxidation, inflammation, intracellular calcium overload, and irreversible apoptosis/necrosis—collectively termed MIRI [3]. The core distinction lies in their pathogenesis: MIRI represents additional damage from ischemia-reperfusion (IR) processes, whereas MI stems from direct injury due to persistent ischemia. Thus, MIRI primarily involves early explosive inflammation and secondary injury upon reperfusion [4], while MI entails a dynamic interplay of acute inflammation followed by tissue repair and fibrosis [5].

The application of technologies such as extracorporeal circulation in cardiac surgery has rendered MIRI a significant challenge that undermines the efficacy of cardiovascular surgical procedures. However, the mechanisms underlying reperfusion injury are still not fully understood, encompassing molecular, cellular, and tissue-level changes. This review explores the macrophage-mediated immune and inflammatory responses during MIRI and MI.

## 2. Phagocytosis and Digestion of Macrophages

**Figure 1:**
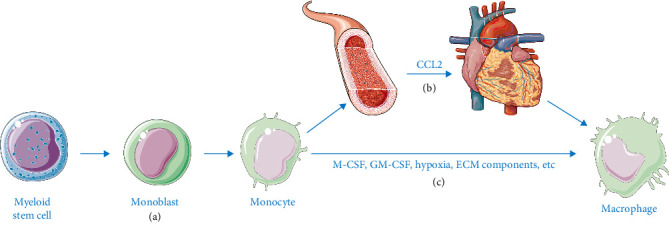
The recruitment of macrophages. (a) Bone marrow stem cells differentiate into monoblasts, which in turn produce monocytes. (b) Mononuclear cells are transported to the heart via the peripheral blood and differentiate into macrophages. (c) These monocytes are released from the bone marrow into the peripheral blood, where they are stimulated and activated by cytokines such as macrophage-colony stimulating factor (M-CSF) and granulocyte-macrophage colony stimulating factor (GM-CSF), as well as by environmental factors like hypoxia and extracellular matrix (ECM) components. This process transforms them into macrophages.

Phagocytosis is essential for maintaining tissue homeostasis and defending against invasive pathogens. However, it may also disrupt innate immunity [6]. Derived from bone marrow monocytes, macrophages migrate to damaged sites via chemotactic signals during infection or injury. They ingest pathogens, debris, and parasites through phagolysosomal digestion [7]. By recognizing apoptotic markers (e.g., phosphatidylserine) via surface receptors (triggering receptor expressed on myeloid cells 2 [TREM2], CD36), macrophages clear necrotic cardiomyocytes and debris, thereby preventing secondary damage [8]. Additionally, they present antigens to activate other immune cells. Macrophages consist of both tissue-resident and recruited subsets. Following ischemia, necrotic cells release damage-associated molecular patterns (DAMPs) (e.g., high mobility group protein [HMGB]1, adenosine triphosphate [ATP]), which activate pattern recognition receptors to recruit monocytes to infarcted areas [9]. Recruited monocytes differentiate into primordial macrophages (Figure 1), while C─C motif ligand (CCL) 2 binding to C─C chemokine receptor type 2 (CCR2) drives monocyte migration and macrophage differentiation [10]. Macrophages are classified as either pro-inflammatory M1 macrophages or reparative M2 macrophages [11]. The polarization dynamics of macrophages in both MIRI and MI are critically regulated by the cytokine milieu. While phagocytic clearance of necrotic cardiomyocytes is fundamental to both conditions, MIRI is characterized by acute oxidative stress-triggered recruitment, whereas MI involves sustained DAMP-driven monocyte infiltration alongside progressive tissue remodeling.

## 3. Macrophage Behaviors in MIRI and MI by Immunity and Inflammation

### 3.1. Polarization of Mononuclear-Macrophage in MIRI and MI

**Figure 2:**
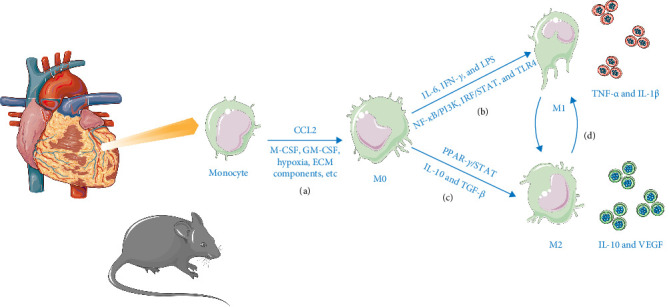
Role of macrophage polarization regulatory network in MIRI and MI. (a) Monocytes are recruited into myocardial tissue and differentiate into macrophages following myocardial ischemic injury or infarction. (b) Pro-inflammatory signals (IL-6, IFN-γ, and LPS) activate the M1 phenotype via the NF-κB/PI3K, IRF/STAT, and TLR4 pathways, leading to the secretion of factors such as TNF-α and IL-1β, thereby establishing a positive feedback loop of inflammation. (c) Anti-inflammatory signals (IL-10 and TGF-β) induce the M2 phenotype via the PPAR-γ/STAT pathway, leading to the secretion of repair factors such as IL-10 and VEGF, thereby establishing a positive feedback loop of anti-inflammation. (d) M1 and M2 macrophages demonstrate plasticity, allowing for dynamic interconversion.

Pro-inflammatory factors such as interleukin 6 (IL-6) and interferon-gamma (IFN-γ) drive M1 polarization via the nuclear factor kappa-B (NF-*κ*B) /phosphatidylinositol (PI) 3-kinase, interferon regulatory factor (IRF) /signal transducer and activator of transcription (STAT), and lipopolysaccharide (LPS) /toll-like receptor 4 (TLR4) pathways [12, 13]. Activated M1 macrophages secrete additional inflammatory mediators upon receptor engagement (Figure 2). Cardiomyocyte-derived cytokines and factors secreted by M1 macrophages create an inflammatory microenvironment [14] that propels primordial macrophages toward M1 polarization. This process forms a pro-inflammatory positive feedback loop, exacerbating inflammation and myocardial damage.

Conversely, anti-inflammatory factors, including IL-10 and transforming growth factor-β (TGF-β), promote M2 polarization. However, disruptions in cytokine balance—such as excessive pro-inflammatory signals or deficient anti-inflammatory signaling—can impede the transition to the M2 phenotype. Transcription factors induce M2-specific gene expression through peroxisome proliferator-activated receptor γ (PPAR-γ) and STAT pathways, with additional contributions from the local microenvironment, apoptotic debris, extracellular matrix (ECM) components, and specific immune subsets. During MIRI, M2 macrophages exert anti-inflammatory effects, and their secreted factors further polarize other macrophages toward the M2 phenotype [13]. The factors secreted by these macrophages further polarize other macrophages toward the M2 phenotype, thereby establishing anti-inflammatory positive feedback loops that reduce inflammation and provide cardiac protection.

The M1/M2 classification offers a foundational framework, yet it falls short in fully capturing the complex heterogeneity of macrophages in MIRI, particularly with respect to the detailed characteristics and functions of distinct macrophage subgroups [15]. During myocardial injury, macrophages display dynamic plasticity, enabling differentiated M1 macrophages to transition into M2 phenotypes [16]. In MIRI, ROS burst upon reperfusion sustains M1 dominance, delaying M2 transition and amplifying secondary injury. In MI, DAMPs from persistent ischemia gradually shift macrophages toward TREM2^high^ repair phenotypes to resolve inflammation [5]. This polarization dynamic is observed in both MIRI and MI. In MIRI, acute oxidative stress drives a predominance of M1 polarization early on, whereas in MI, sustained inflammatory signaling persists throughout the tissue remodeling phases [17].

### 3.2. Tissue-Resident Macrophages

Classic cardiac tissue-resident macrophages express markers such as LYVE1, GAS6, and CBR2 [18], which enable them to sense cytokines from injured myocardium and stimulate angiogenesis while preventing fibrosis [19]. During MI, CCR2^high^ and TREM2^high^ macrophages emerge as major subsets in the early and late phases, respectively [20]. CCR2 expression levels dictate functional diversity. Embryonic-derived CCR2^−^ macrophages lack inflammatory markers and confer postischemic protection [21], whereas monocyte-derived CCR2^+^ counterparts slowly replenish over time [22], expressing inflammatory factors (e.g., TNF and chemokines) and activating pathways like MYD88 to produce monocyte-recruiting chemokines (CCL2/MCP1 and CCL7/MCP3) [18]. In contrast, resident CCR2^−^ macrophages inhibit monocyte recruitment [23]. Further stratification of CCR2 expression reveals that CCR2^low^ macrophages in early injury express proliferation and self-renewal genes (e.g., BIRC5, STMN1, UBE2C, and TOP2A) [24] and exhibit enhanced efferocytosis compared to recruited CCR2^+^ macrophages [25]. Later, angiogenesis-associated genes (e.g., FN1, SLC7A2, and SDC3) dominate [22]. Additionally, transient BHLHE41^+^ macrophages, which peak in developing infarcts, secrete granulin to antagonize TNF-α/TNFR1 and reduce myofibroblast activity [26]. Conversely, fibrosis genes (e.g., BGN, COL1A2, DCN, COL1A1, and COL3A1) contribute to tissue scarring [27].

In the context of MIRI, the acute oxidative burst upon reperfusion dominantly activates CCR2^high^ macrophages, driving rapid M1 polarization and amplifying inflammation through ROS-mediated DAMP release (e.g., HMGB1 and ATP). This contrasts with MI, where TREM2^high^ macrophages gradually dominate to coordinate prolonged angiogenesis-fibrosis balance during remodeling [17]. Although these macrophages are not involved in initial infarct size, they critically modulate post-MI ventricular remodeling [23], with tissue-specific populations impacting cross-organ injury repair [28]. While resident macrophages participate in both MIRI and MI, their roles diverge. In MIRI's acute phase, they primarily mitigate oxidative stress and limit secondary necrosis, whereas, in MI, they orchestrate a sustained balance between angiogenesis and fibrosis during prolonged remodeling.

### 3.3. Monocytes and Recruit Macrophages

**Figure 3:**
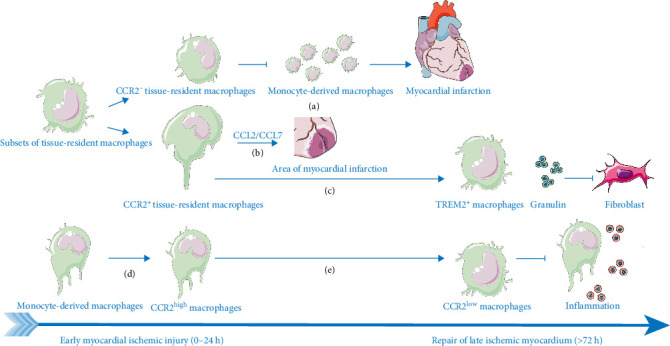
Spatiotemporal dynamics of tissue resident and monocyte-derived macrophages. (a) Embryonically derived CCR2-negative cells exhibit high expression of LYVE1 and GAS6, which inhibits monocyte recruitment and confers early protective effects. (b) The mononuclear CCR2^+^ source secretes CCL2/CCL7 to promote inflammation. (c) The CCR2^+^ mononuclear cells differentiate into the TREM2^+^ subpopulation during the later stages, secreting granulin to inhibit fibrosis. (d) Monocyte-derived macrophages are differentiated into those with high CCR2 expression. (e) The early CCR2^high^ macrophages transition to the late CCR2^low^ macrophages.

Injured myocardium recruits bone marrow-derived monocytes into circulation via tissue-resident macrophages, with monocytes detectable by markers LY6C2 and PLAC8. Monocyte infiltration initiates through CCL2/CCR2 signaling and CX3CR1 expression [23], differentiating into pro-inflammatory macrophages that replace resident populations early post-injury. These recruited macrophages express elevated S100A8, S100A9, PF4, CCL7, and FABP5 [29], facilitating leukocyte chemotaxis while aggravating damage via inflammatory secretion yet enabling necrotic cell clearance. Pro-inflammatory macrophages predominantly express CCR2 and MHC-II, with functional heterogeneity linked to CCR2 levels (Figure 3): Recruited CCR2^+^ macrophages exhibit higher inflammatory gene expression (enriched in TNF/NF-κB/RAS/IL2 pathways), arginase 1, MRC1, and HIF1α versus tissue-resident CCR2^+^ subsets [23]. CCR2^high^ macrophages peak during acute inflammation [20], uniquely expressing RSAD2 [30] and secreting meteorin-like (METRNL) secretory protein to promote tissue protection/metabolic adaptation [31, 32] via KIT receptor-mediated angiogenesis and repair [33]. Critically, in MIRI, recruited CCR2^high^ macrophages rapidly secrete METRNL to mitigate acute oxidative injury and enhance phagocytic clearance of necrotic cells within hours of reperfusion. Conversely, in MI, the same subset primarily orchestrates TGF-β/VEGF-driven fibrosis over weeks of remodeling [4]. CCR2^low^ macrophages express anti-inflammatory GPNMB and HMOX1 [24, 34]. MHC-II^high^ macrophages drive inflammatory polarization, secrete IL-1β, clear apoptotic cells via AXL [35], and participate in antigen presentation, while MHC-II^low^ subsets enhance phagocytosis of necrotic cardiomyocytes early post-MIRI [36]. CD72^high^ macrophages (Rel-regulated) exhibit inflammatory phenotypes with elevated IL6/TNF [37]. Post-acute inflammation, recruited TREM2^+^ macrophages (predominantly CCR2^+^MHCII^low^) surpass resident TREM2^+^ cells in importance, secreting itaconate to inhibit cardiomyocyte apoptosis and promote fibroblast proliferation [35, 38]. TREM2^high^ macrophages (monocyte-derived) increase numerically with low FOLR2/LYVE1/MGL2 expression, displaying dominant anti-inflammatory traits. Soluble TREM2 modulates polarization, and TREM2 deficiency impairs endocytosis while inhibiting SLC25A53 via SYK-SMAD4 post-efferocytosis [35, 38]. Notably, CCL2-activated CCR2^+^ macrophages transform into repair phenotypes secreting TGF-β and VEGF to promote fibrosis [39]. While monocyte-derived macrophage recruitment occurs in both MIRI and MI, their functional emphasis differs. In MIRI, they primarily mitigate acute oxidative injury through rapid METRNL secretion and phagocytic clearance, whereas, in MI, they orchestrate sustained fibrotic remodeling via TGF-β/VEGF-driven mechanisms.

### 3.4. Communication Between Macrophages and Other Immune Inflammatory Cells

**Figure 4:**
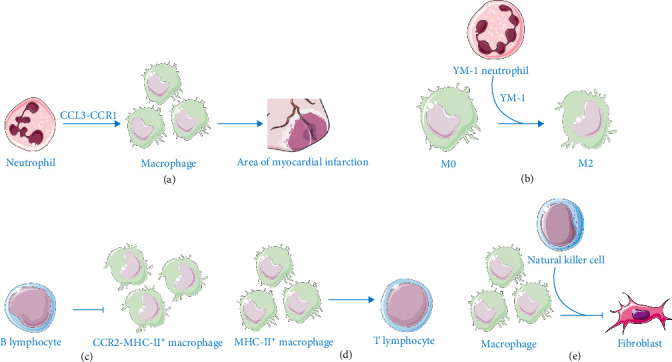
Crosstalk between macrophages and other immune cells. (a) Neutrophils recruit macrophages into the infarcted myocardium via CCL3-CCR1 signaling. (b) YM-1^+^ neutrophils induce M2 macrophage polarization by YM-1. (c) The number of CCR2-MHC-II^high^ macrophages is regulated by B cells through an unknown mechanism. (d) MHC-II^+^ macrophage antigen presentation activates regulatory T cells. (e) NK cells secrete proangiogenic factors, which collaborate with macrophages to inhibit fibrosis.

In the early phase of MIRI, monocyte-independent macrophages alter immune crosstalk and induce pro-inflammatory neutrophil polarization [40]. Validated macrophage–neutrophil ligand-receptor pairs, including IGF1-IGF1R and colony stimulating factor (CSF)1-CSF1R [41, 42], exhibit high expression [43]. Tissue-resident macrophages recruit CCL3^high^ neutrophils, which subsequently mediate inflammatory macrophage recruitment [43]. Prolonged IR triggers peak cytokine and chemokine secretion, coinciding with maximal macrophage and NK cell infiltration [44]. Post-acutely, neutrophils regulate macrophage function to promote repair (Figure 4). Neutrophil-derived YM-1 protects against MIRI by driving M2 polarization [43, 44]. Recruited macrophages regulate T cell activation, characterized by high expression of CD209D, DPP-4, and H_2_O_5_ [18]. In later injury stages, monocyte–macrophage functions and T cell differentiation are modulated by lncRNA NEAT1 [45]. CD4^+^ T cells recognize cardiac antigens via dendritic cells (DCs), differentiating into Tregs to resolve inflammation through reduced apoptosis and enhanced cardiomyocyte proliferation [46]. MHC-II^+^ macrophages and DCs engage T cells via antigen presentation, influencing inflammation, repair, and remodeling [47]. Treg-derived IL-35 activates macrophage CX3CR1 and TGF-β1 through GP130 signaling [48]. B cells adjacent to macrophages modulate myocardial CCR2-MHC-II^high^ populations [49], while NK cells prevent fibrosis and promote angiogenesis/vascularization during MI repair [50].

These immune interactions occur in both MIRI and MI, but their temporal dominance differs: Neutrophil–macrophage crosstalk drives acute inflammation resolution in MIRI, whereas T/B cell-mediated regulation orchestrates chronic remodeling in MI.

## 4. Macrophage-Associated Therapeutic Targets

### 4.1. Anti-Inflammatory Therapy

Anti-inflammatory therapy may mitigate the progression of MIRI. Early monocyte recruitment can be suppressed by targeting *G* protein-coupled receptor kinase 5 [51]. Conversely, neogenin deficiency exacerbates macrophage infiltration and pro-inflammatory polarization via the JAK1-STAT1 pathway [52]. MI impairs the biosynthesis of 5-methoxytryptophan (5-MTP), an anti-inflammatory and anti-fibrotic agent that reduces T cell and macrophage infiltration [53]. S100A9 promotes repair by driving monocyte dynamics and generating reparative LY6C^low^MERTK^high^ macrophages [29]. After infiltration, I*κ*B kinase protects against MIRI by negatively regulating M1 polarization [54]. Smad3 mediates the transition of macrophages to anti-inflammatory phenotypes; Smad3^null^ macrophages exhibit reduced expression of TGF-β1, IL-10, angiogenic factors, and phagocytosis-related milk fat globule EGF factor-8 [55]. Alpha-lipoic acid (ALA) suppresses hypoxic inflammatory cytokines, promotes M2b polarization, and inhibits HMGB1/NF-*κ*B in macrophages. Supernatants containing ALA block apoptosis and autophagy in hypoxic cardiomyocytes [56]. PCSK9 modulates macrophage polarization-mediated post-MI remodeling [57]. Galectin-3^high^CD206^+^ macrophages produce osteopontin (OPN), whose polarization via the IL-10-STAT3-Galectin-3 axis promotes fibrosis and apoptotic clearance [58]. CARD9 in F4/80^+^ macrophages mediates pro-inflammatory cascades, potentially driving adverse remodeling through NF-κB-regulated lipocalin 2 [59].

These targets hold translational potential for both MIRI and MI, yet they require distinct intervention strategies. MIRI therapies should prioritize acute-phase inflammation suppression (e.g., I*κ*B kinase modulation), while MI interventions must balance fibrosis regulation (e.g., SMAD3/OPN targeting) with chronic inflammation control.

### 4.2. Extracellular Vesicles (EVs)

Exosomes, secreted by cardiomyocytes, mesenchymal stem cells (MSCs), and cardiac progenitor cells, are EVs rich in miRNA, mRNA, proteins, and lipids [60]. These vesicles regulate macrophage function. Exosomal markers such as CD47 and integrins evade mononuclear phagocytic clearance and enable macrophage targeting, which is crucial for treating myocardial ischemic injury [61]. Exosome-carried miRNAs modulate macrophage polarization in several ways. MiR-21 activates STAT3 to induce M2 polarization, which has anti-inflammatory and repair effects [62]; miR-155 inhibitors reduce TNF-α and IL-6 by blocking the NF-*κ*B/JNK pathways [63]; miR-30e targets NLRP3 to suppress the secretion of IL-1β and IL-18 [64, 65]. Exosomes also deliver anti-inflammatory cytokines such as TGF-β and IL-10 to inhibit M1 phenotypes. Beyond polarization, exosomes upregulate macrophage MERTK receptors to enhance phagocytosis and debris clearance [66], while miR-29b reduces fibrosis by downregulating collagen genes (COL1A1 and COL3A1) [67]. Collectively, these mechanisms suppress inflammation, promote repair, and modulate fibrosis post-injury.

While exosomal therapies offer benefits for both MIRI and MI, their application should be temporally optimized. Acute MIRI requires rapid anti-inflammatory delivery (e.g., NLRP3-targeting miR-30e), whereas chronic MI demands sustained antifibrotic effects (e.g., collagen-suppressing miR-29 b).

### 4.3. Stem Cell Therapy

Stem cell therapy improves cardiac function through acute sterile immune responses, characterized by CCR2^+^ and CX3CR1^+^ macrophages that reduce ECM in border zones by modulating fibroblast activity [68]. Cortical bone stem cells (CBSCs) secrete TGF-β and macrophage colony-stimulating factor (M-CSF), influencing cardiomyocyte and non-cardiomyocyte cell death as well as immune cell recruitment [69]. CBSC therapy increases the number of galectin-3^+^ macrophages, fibroblasts, and CD4^+^ T cells while reducing cardiomyocyte apoptosis. In CBSC-conditioned medium, macrophages polarize toward anti-inflammatory phenotypes, secreting IL-10, TGF-β, and IL-1RA with enhanced endocytosis [70]. CBSC-derived EVs (CBSC-EVs) drive macrophage polarization and proreparative T cell phenotypes. Specifically, miR-182/183-enriched EVs mediate metabolic reprogramming and reparative polarization through the RASA1 axis under LPS stimulation [71]. MSCs regulate the balance of bone marrow-derived M1 and M2 macrophages [72] and decrease neutrophil numbers by enhancing M2 macrophage-mediated efferocytosis [73].

While both MIRI and MI benefit from stem cell-mediated macrophage modulation, the therapeutic focus differs based on the injury phase. Acute MIRI requires neutrophil-focused interventions, where MSCs offer an advantage, whereas chronic MI demands fibrotic regulation, where CBSCs and CBSC-EVs demonstrate superiority.

## 5. Advances in Clinical Research on Anti-Inflammatory Cytokine-Targeted Therapy

The core objective of clinical translation is to enhance post-injury repair by suppressing pro-inflammatory macrophages while promoting reparative subsets, primarily through targeted anti-inflammatory cytokine therapy. The CANTOS trial demonstrated that Canakinumab (anti-IL-1β) significantly reduces post-MI cardiovascular event recurrence [74, 75], validating IL-1β blockade as an effective strategy for improving MI prognosis. Similarly, Anakinra (IL-1 receptor antagonist) improved cardiac function in MI patients according to the VCU-ART trials [76], reduced new-onset heart failure hospitalizations in STEMI patients after 14-day therapy [77], and attenuated systemic inflammation in STEMI [78]. Tocilizumab (anti-IL-6R) enhanced myocardial salvage in acute STEMI [79].

These strategies effectively modulate macrophage phenotypes, inhibit inflammation, and promote repair. While cytokine-targeting therapies show promise for both MIRI and MI, the therapeutic focus differs based on the injury phase. MI management benefits from chronic IL-1/6 pathway inhibition (e.g., Canakinumab), whereas MIRI requires acute-phase interventions (e.g., short-course Anakinra in reperfusion settings). Long-term efficacy and safety of these therapies require further evaluation.

## 6. Conclusion

CAD constitutes the fundamental pathological basis for myocardial ischemic injury and MI, while myocardial reperfusion—though clinically essential for salvage—paradoxically triggers MIRI. Our synthesis demonstrates that macrophage heterogeneity and plasticity critically dictate divergent pathological outcomes. In MIRI, acute oxidative stress traps CCR2hi macrophages in a pro-inflammatory state, exacerbating secondary damage through ROS–DAMP cascades, whereas, in MI, phased CCR2^high^-to-TREM2^high^ macrophage transition dynamically orchestrates repair-fibrosis balance during chronic remodeling. Furthermore, we decode spatiotemporal immune crosstalk networks—notably neutrophil–macrophage axis dominance in MIRI's hyperacute phase versus B cell–macrophage axis regulation in late MI—and advance a transformative therapeutic framework spanning molecular targeting (e.g., IL-1β/IL-6 blockade), precision delivery systems (phase-optimized nanocarriers/hydrogels), and regenerative engineering (stem cell–exosome reprogramming). Collectively, these insights position spatiotemporally precise macrophage-centered modulation as the paradigm to halt injury progression and restore cardiac homeostasis.

## 7. Discussion

The conventional M1/M2 classification of macrophages falls short in comprehensively defining their roles in cardiac injury particularly in distinguishing acute reperfusion injury (MIRI) from chronic infarction (MI) [11], thus necessitating advanced characterization via single-cell sequencing. This approach reveals subset heterogeneity based on spatiotemporal dynamics, injury progression, and metabolic states [18, 80]. In MIRI, CCR2^high^RSAD2^+^ macrophages dominate peri-infarct zones within 6 h, driving ROS amplification via NOX2 upregulation [4]. In MI, TREM2^high^FOLR2^low^ macrophages expand in fibrotic borders at Day 7, secreting PDGF-BB to activate fibroblasts [20]. This spatial–temporal resolution demonstrates that macrophage heterogeneity is injury-phase-dependent, challenging the static M1/M2 paradigm. During myocardial injury, macrophages exhibit remarkable plasticity. Monocyte-derived subsets transition from early pro-inflammatory phenotypes, which are essential for necrotic clearance, to late reparative states. Crucially, plasticity dynamics diverge in MIRI vs., MI. In MIRI, reperfusion-induced ROS burst locks monocyte-derived macrophages in a pro-inflammatory state via HIF-1α/NF-κB coactivation, delaying M2 transition >72 h and amplifying cardiomyocyte ferroptosis [4]; in MI, hypoxic gradients in persistent ischemia gradually polarize macrophages toward TREM2^high^ phenotypes through AMPK/PGC-1α-mediated metabolic reprogramming, enabling phased repair [38]. This explains why MIRI therapies require acute inflammation interception, while MI demands sustained modulation of macrophage metabolism. Meanwhile, resident macrophages demonstrate context-dependent functional adaptations. This phenotypic flexibility, influenced by cellular origin, highlights the paradoxical importance of controlled inflammation in initiating MI repair.

However, critical knowledge gaps impede therapeutic advancement. Emerging evidence reveals phase-specific immune crosstalk wherein neutrophil-derived YM-1 instructs macrophage M2 polarization via IL-4Rα/JAK3-STAT6 signaling during early MIRI (0–24 h), but reperfusion-generated ROS oxidizes YM-1, blunting this protective axis and perpetuating inflammation [43]; conversely, in late MI (Day 14+), cardiac-associated B cells sustain TREM2^high^ macrophage survival through APRIL/TACI signaling, directly promoting collagen crosslinking and fibrosis progression [49]. This temporal hierarchy underscores that neutrophil–macrophage crosstalk is therapeutically targetable only within the hyperacute phase of MIRI, whereas B cell–macrophage interactions dominate chronic MI remodeling—highlighting the need for precision timing in interventions. Nevertheless, fundamental questions remain unresolved regarding how B cells, T cells, NK cells, and DCs collectively orchestrate macrophage function across acute inflammation and chronic remodeling phases [50].

Current therapies targeting macrophage regulation, such as IL-1 blockade [74], engineered exosomes, and emerging approaches like pericardial GATA6^+^ macrophage modulation [81] or cholinergic immunomodulation [82], show efficacy but face translational limitations including spatial specificity challenges, cost-efficacy barriers, and inadequate temporal precision. To overcome these spatiotemporal constraints, next-generation strategies must embrace phase-specific delivery systems—such as nanoparticles releasing IL-1β siRNA within 2 h of reperfusion to block NLRP3 inflammasome in CCR2^high^ macrophages during MIRI [4] and hydrogel scaffolds slowly releasing METRNL to polarize TREM2^high^ macrophages in fibrotic zones of chronic MI [33]. Concurrently, microenvironmental reprogramming via BDNF-functionalized patches enhances reparative macrophage survival in border zones [83] but necessitates hypoxia-responsive vectors to prevent off-target effects in normoxic MIRI myocardium. Further leveraging single-cell-guided precision, spatial transcriptomics identifies CCR2^high^PF4^+^ macrophages as key drivers of MIRI arrhythmogenesis [4], enabling subtype-specific ablation. Collectively, such innovations will shift macrophage-targeted therapies from broad suppression to context-aware restoration of the inflammation-repair equilibrium, thereby transforming therapeutic efficacy and specificity.

## Data Availability

The authors have nothing to report.
